# Supplementary material for: Loneliness during a strict lockdown: Trajectories and predictors during the COVID-19 pandemic in 38,217 United Kingdom adults
Source: Soc Sci Med. 2020 Nov;265:113521. doi: 10.1016/j.socscimed.2020.113521 (PMC7768183; doi:10.1016/j.socscimed.2020.113521)
Supplement: Multimedia component 1 [file mmc1.pdf]

Supplementary Material

Table S1: Comparison of items in the original and revised Perceived Social Support Questionnaire (F-SozU K-6).

**Original**	**Adapted for COVID-19**<br>**In the past week, I feel…**
I experience a lot of understanding and security from others	I have experienced a lot of understanding and support from others
I know a very close person whose help I can always count on	I have a very close person whose help I can always count on
If necessary, I can easily borrow something I might need from neighbours or friends	If necessary, I can easily borrow something I need from neighbours or friends
I know several people with whom I like to do things	I have people with whom I can spend time and do things together
When I am sick, I can without hesitation ask friends and family to take care of<br>important matters for me	If I get sick, I have friends and family who will take care of me
If I am down, I know to whom I can go without hesitation	If I am feeling down, I have people I can talk to without hesitation

Table S2 Descriptive statistic of the explanatory variables (*N*=38,217, weighted)

Variables		Percentages
Age	18-29	19.50%
	30-45	26.10%
	46-59	24.10%
	60+	30.30%
Gender	Women (vs. men)	50.60%
Ethnicity	Non-white (vs. white)	12.80%
Education	GCSE or below	32.70%
	A-levels or equivalent	33.90%
	Degree or above	33.41%
Household income	Low (<30k) (vs. high)	47.60%
Employment status	Employed	57.68%
	Unemployed	2.63%
	Student	7.97%
	Inactive other	31.71%
Mental health	Diagnosed condition	19.86%
Living status	Living with others (vs. alone)	81.33%
Area of living	Rural (vs. urban)	20.49%
Number of close friends	Large (>=3) (vs. small)	69.61%
Usual face-to-face contact	At least weekly (vs. < weekly)	67.78%
Perceived social support	High (sum score >=18) (vs. low)	73.28%

Table S3 Estimated odds ratios, standard errors, *p* values of the predictors of latent growth trajectory classes, with interaction terms (*N*=38,217)

Variables		Med-low (vs. lowest) LC2 (vs. LC1)			Med-high (vs. lowest) LC3 (vs. LC1)			Highest (vs. lowest) LC4 (vs. LC1)		
		OR	*SE*	*p*	*OR*	*SE*	*p*	*OR*	*SE*	*p*
Age	18-29	2.57	0.43	0.000	3.36	0.45	0.000	5.94	0.94	0.000
	30-45	1.54	0.18	0.003	2.51	0.22	0.000	3.94	0.44	0.000
	46-59	1.11	0.11	0.322	1.68	0.13	0.000	2.05	0.20	0.000
	60+	Ref	Ref.	Ref.	Ref	Ref.	Ref.	Ref	Ref.	Ref.
Gender	Women (Ref. men)	1.37	0.11	0.001	1.48	0.09	0.000	1.70	0.14	0.000
Ethnicity	Non-white (Ref. white)	1.28	0.23	0.220	1.06	0.14	0.687	0.96	0.16	0.823
Education	GCSE or below	Ref	Ref.	Ref.	Ref	Ref.	Ref.	Ref	Ref.	Ref.
	A-levels or equivalent	1.07	0.11	0.560	1.11	0.09	0.218	1.09	0.11	0.415
	Degree or above	1.00	0.10	0.974	1.02	0.08	0.847	0.86	0.09	0.119
Household income	Low (<30k) (Ref. high)	1.00	0.09	0.985	1.23	0.09	0.006	1.33	0.12	0.006
Employment status	Employed	Ref	Ref.	Ref.	Ref	Ref.	Ref.	Ref	Ref.	Ref.
	Unemployed	1.25	0.30	0.402	1.32	0.29	0.269	1.75	0.41	0.064
	Student	1.36	0.36	0.315	1.44	0.29	0.125	2.14	0.47	0.016
	Inactive other	0.98	0.10	0.818	0.94	0.07	0.387	1.22	0.11	0.048
Mental health	Diagnosed condition	1.41	0.51	0.424	1.79	0.49	0.108	4.49	1.26	0.006
Living status	Living with others (Ref. alone)	0.44	0.05	0.000	0.40	0.03	0.000	0.26	0.03	0.000
Area of living	Rural (Ref. urban)	0.91	0.08	0.265	0.87	0.06	0.022	0.73	0.07	0.000
Number of close friends	Large (>=3) (Ref. small)	1.02	0.10	0.875	0.83	0.06	0.007	0.61	0.06	0.000
Usual face-to-face contact	At least weekly (Ref. < weekly)	0.88	0.08	0.136	1.20	0.08	0.017	1.13	0.10	0.220
Perceived social support	High (sum score >=18) (Ref. low)	0.54	0.06	0.000	0.24	0.02	0.000	0.11	0.01	0.000
Living status	* Mental health	1.20	0.32	0.531	1.65	0.34	0.058	1.04	0.23	0.859
Area of living	* Mental health	1.08	0.31	0.788	0.96	0.19	0.836	1.17	0.27	0.528
Number of close friends	* Mental health	0.83	0.20	0.394	0.96	0.18	0.800	0.83	0.17	0.322
Usual face-to-face contact	* Mental health	1.35	0.30	0.245	0.89	0.15	0.451	0.99	0.19	0.949
Perceived social support	* Mental health	1.05	0.26	0.859	1.26	0.24	0.276	1.17	0.24	0.484

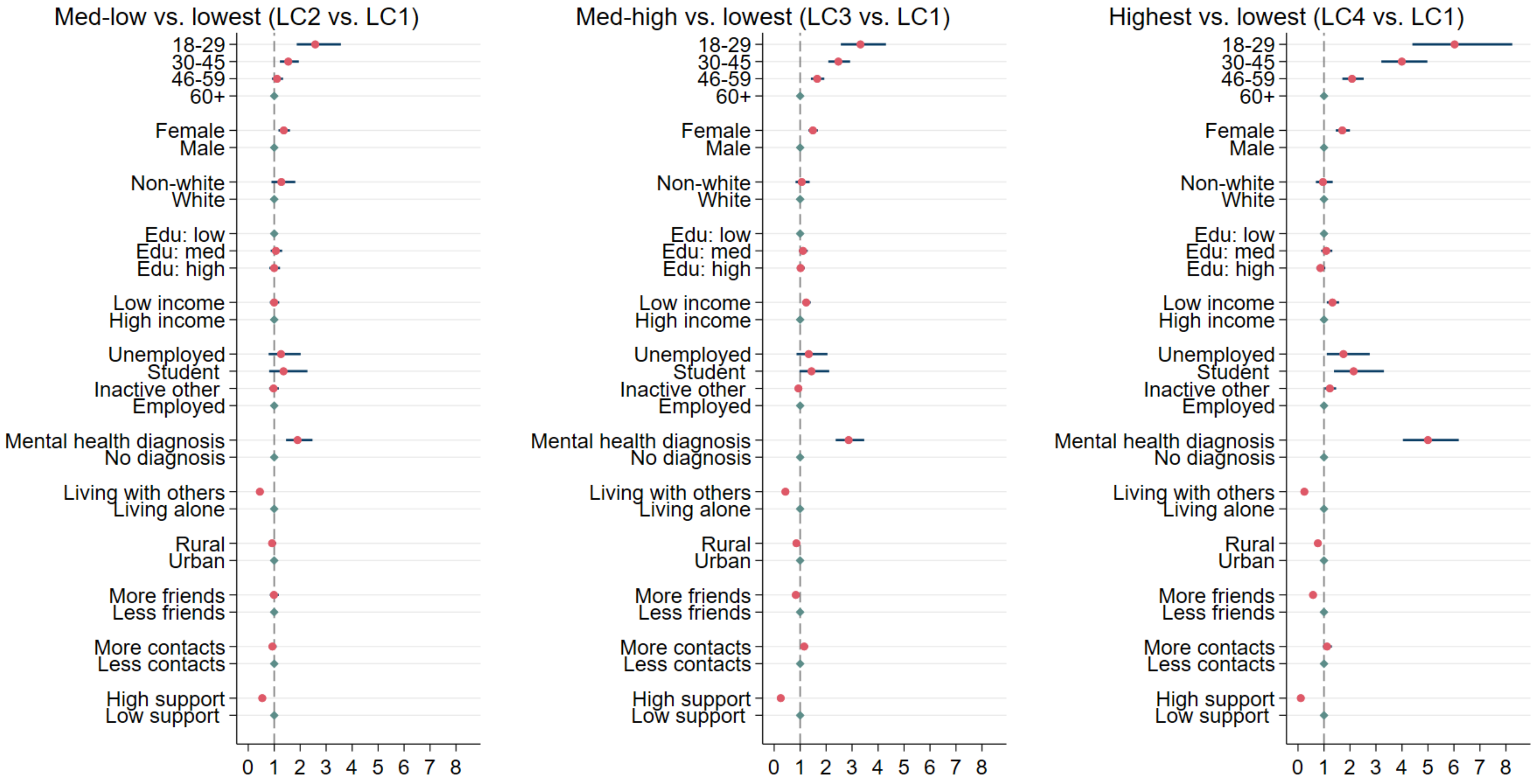

Figure S1. Estimated odds ratios and 95% confidence intervals for the predictors of latent growth trajectory classes (Table 2)
